# Anæsthetics: A Stimulus to Voluntary Effort

**Published:** 1887-04-23

**Authors:** 


### ANÆSTHETICS: A STIMULUS TO VOLUNTARY EFFORT.

By a Lady of Title.

I should like to offer a suggestion, *in case* it should be at all worth considering, in connection with the insolvent state of the hospitals, and the desirability of stimulating voluntary effort on their behalf. It seems to me that if all who have been spared suffering, or have seen any they loved spared suffering, of any kind or degree, through the administration of anæsthetics, would testify their gratitude by a thank-offering of from 1*d.* to £5, a goodly sum might be raised for the relief of the hospitals. I think the incentive ought to prove a strong one, the area of the appeal would be wide yet definite, and the application of the funds appropriate. Of course, this offering should not check in any way the hoped-for tide of general subscriptions during the Hospitals Fortnight; but I think many persons who might be indifferent to a general appeal will respond more readily to a special one made to them, as persons belonging to a particular category such as that I am suggesting. It would be a large one, including so many beyond the army of invalids. Why, the dentist's patients alone might offer a substantial tribute! Surely it is *right* that so great a gift from God to man as the gift of anæsthetics, bestowed through the medium of human intelligence, should receive some sort of national recognition! Perhaps the general appeal and the special one might be made concurrently. But I have said enough about a proposal which may, after all, be of no practical value whatever. (M. G.)

